# The Leverage Effect on Wealth Distribution in a Controllable Laboratory Stock Market

**DOI:** 10.1371/journal.pone.0100681

**Published:** 2014-06-26

**Authors:** Chenge Zhu, Guang Yang, Kenan An, Jiping Huang

**Affiliations:** Department of Physics and State Key Laboratory of Surface Physics, Fudan University, Shanghai, China; Universidad Veracruzana, Mexico

## Abstract

Wealth distribution has always been an important issue in our economic and social life, since it affects the harmony and stabilization of the society. Under the background of widely used financial tools to raise leverage these years, we studied the leverage effect on wealth distribution of a population in a controllable laboratory market in which we have conducted several human experiments, and drawn the conclusion that higher leverage leads to a higher Gini coefficient in the market. A higher Gini coefficient means the wealth distribution among a population becomes more unequal. This is a result of the ascending risk with growing leverage level in the market plus the diversified trading abilities and risk preference of the participants. This work sheds light on the effects of leverage and its related regulations, especially its impact on wealth distribution. It also shows the capability of the method of controllable laboratory markets which could be helpful in several fields of study such as economics, econophysics and sociology.

## Introduction

Nowadays, wealth distribution has always been a heated and vital issue in economics, since it greatly concerns the happiness and stabilization of populations in different countries. If the wealth or income gap in a country or region between the rich and the poor is too wide, it may cause many economic and social troubles. So it has drawn great attention from not only the academic side, but also the governmental side in various countries. Definitely, wealth distribution is a very general topic which is determined by many factors. However, with the background of the recent financial crisis, in which innovative financial tools have been used frequently to raise leverage, we want to specifically study how the wealth distribution is affected by the usage of leverage in capital markets, such as stock markets.

As we know, leverage is a general term for any tool or technique that is used to amplify gains and losses, most often it is in the form of buying more of an asset by using borrowed funds (That’s what we will focus on in this work). The belief is that when utilized in the right time and right way, it will multiply the profit from investing, which is quite attractive to participants in financial markets. Up to date, leverage has played an increasingly important role in both developed and emerging markets. With its growing usage in economic activities, it has assisted the investors to manage their wealth with more resources. Meanwhile, it has also attracted many arguments around its effects on financial markets. While some believe that it is a positive tool for gaining without abundant resources, and a powerful financing source for investors especially qualified companies to compete [1]–[4]; others reckon that the overused leverage will lead to a more unequal wealth distribution among participants, as well as more fluctuations and worse instability in the markets [5]–[8], [20]–[23]. Here, one important aspect of its effects that we want to study is on wealth distribution, since it is not only a direct result of leverage usage on the participants in markets, but also a key issue of resources allocation, which is a fundamental subject of econophysics through its development [9]–[16]. Besides, we have also developed a proper tool for the study: a controllable laboratory market, which has been already proved efficient in study of econophysics [15]–[19].

From the positive view, leverage is treated as an effective tool for controlling financial crises. In the work of Feldman [1], he recognized the merits of leverage in the regime with share restriction by using an agent-based model to simulate the effects of regulations of financial leverage in a stock market containing one stock, and the result suggested that leverage with proper restriction could lead to less financial crises per century. Besides, leverage is also treated as a useful strategy for competition, especially on the level of corporations. In Hamel’s work [2], the author used the empirical analysis on the performance of different corporations and found that leverage has played as an effective tool for the successful companies to get a larger bang for their buck in the markets, and to allocate their resources more wisely and efficiently. This competition advantage is from the amplification of the investment of corporations, which is just the key feature of leverage, especially for good investors with healthy financial status and wise development strategies, since they can handle the risk and compete in a stable and continuous way.

But when it comes to the leverage effect on wealth distribution, most of the results have been negative. Works on this topic before have mostly used traditional approaches such as empirical analysis and computer modelling to show the relation between changing leverage and wealth distribution. And in most of the works, they drew the conclusion that higher leverage and the inequality of wealth distribution go in the same direction. For example, in the work of Kumhof [20], he used both empirical results and computer model building to study how leverage and crises could rise along with changes in household income distribution. A similar conclusion was drawn in Stockhammer’s study [21] that the polarization of income distribution and the deregulation of financial innovative tools, which mostly used high leverage, were root causes of the recent financial crisis, since they elevated the imbalances that erupted in the crisis. In Blair’s article [22], he stated that the overused leverage and credit caused a more unequal wealth distribution in the United States, since it made the participants in financial markets more vulnerable to any changes in incomes that they were counting on to serve the loans they had taken out. The analysis of empirical data and existing laws were used to show his concerns, and he urged that the regulations on leverage need to be harder. And Agnello [23] studied on the relation between fiscal policies on debt expansion and income inequality by a statistical approach, and reached the conclusion that a sustainable debt path, which would generate a moderate leverage level could help reduce the income inequality.

However, these works have mostly used the traditional ways, and the mechanics behind the positive correlation between leverage and wealth distribution inequality are still not clear. And basically, the nature of wealth distribution is about the allocation of resources, so we choose to adopt our controllable laboratory stock market in this field, which has come out from the classic models of resource allocation such as Minority Game (a.k.a. MG) [9]–[11] and been refined with the flexibility and reliability of the real human players’ participation. This controllable laboratory market method was also successfully used in the studies of another important resource allocation model: Market-Directed-Resource-Allocation-Game (a.k.a. MDRAG) [15], [16], and other related works on capital markets and resource distribution [17]–[19]. Compared to the traditional methods like empirical analysis and computer simulations, the laboratory stock market is a new and insightful tool to study leverage because it introduces the participation of human into an environment of “controlled experiment”. There are two main aspects that make this method meaningful.

First, the “controlled experiment” here means the experiment where one or a few parameters/conditions are purposefully adjusted but all the other parameters/conditions are fixed. This approach directly reveals cause and effect, since it focuses on the main target parameter/condition that is being adjusted and studied. This kind of experiment, which is a classic and vital methodology in both physics and econophyiscs, distinctly differs from the methods of empirical observations. In our market, this adjusted parameter will be the leverage ratio, which represents the magnitude of the leverage in the environment and provides a straightforward cause and effect relation between leverage and wealth distribution. Since in real markets, factors that affect wealth distribution could be various and complex, so with the laboratory market focusing on leverage effect, our work could demonstrate a clear result of the relation between different leverage ratios and wealth distribution.

Second, the introduction of real human participates is also an important feature of the laboratory market, since it makes the market more similar to real ones. And the real process of human beings’ thinking and behaving makes laboratory market more attracting when compared to the artificial market in computer simulations, considering that the intelligence and strategies of computer agents could be somehow unreal or even mistaken. Besides, in our market the incentives of human subjects’ performance are quite equivalent to those in real markets (which will be introduced in the Methods part), hence, the feedback and corresponding behaviors of human subjects could also be expected to be reasonable and reliable. What’s more, the wealth of human subjects in our market is quite clear and traceable, which is an advantage compared to the study focusing on empirical market data, since the situation of wealth distribution in real markets are more complicated and sometimes hard to obtain accurately.

Based on these advantages of the laboratory market, we feel that it would be interesting to study leverage effect with this method to provide some new aspects and thoughts.

In the rest parts of this article, we’ll first demonstrate the properties and mechanics of the controllable laboratory market we used to conduct our human experiments; then show the experimental results and discussion on leverage and wealth distribution.

## Methods

### Ethics statement

In this study, two groups of students from the Department of Physics and School of Economics of Fudan University participated voluntarily. They were all aware of the purpose of scientific research in the experiments, which they were willing to take part in. And all of them have provided their written consent to attend the experiments. This study was approved by the Ethics Committee of Department of Physcis, Fudan University and the whole experiments were conducted under the requirement of ethics.

### The basic conditions of the experiments

Now let’s introduce the laboratory market we used to conduct the human experiments. Generally speaking, we adopted the essential ideas of both financial markets and leverage regulations to build a laboratory market which could reasonably play as the real markets and reproduce the market behavior. To put it briefly, this is a market where one stock can be traded, whose price is determined by supply and demand of players’ orders, just like the real markets; and leverage is available with an adjustable level of allowed leverage ratio and a stable regulation covering qualification criteria and margin calls. The players in the experiments can choose to buy or sell stocks by their own strategies in order to gain profits. Detailed experimental conditions are as below.

We recruited groups of students from Fudan University as volunteers in the experiments to help study the issue. They were from Department of Physics and School of Economics of Fudan University, who attended the experiments voluntarily, and had the necessary knowledge of financial markets and made their trading decisions independently. The experiments were conducted in a computer laboratory of Fudan University. Each participant (player) had a computer to work with, and we would offer all the information about the trading process for them to make decisions. All the computers were linked to an internal local network and a stable web server was set up to handle all the transactions, which ensured the stabilization and fastness in the network. We offered rewards in different forms as incentives to make sure all the participants take part in the experiments with an active attitude of winning, just like reasonable investors in real markets. The details of the rewards will be discussed later.

### The mechanics of trading and pricing

In our market, we set the initial price of the stock 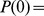
10, and the portfolio of a player consists of two parts: cash and stock shares. 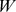, 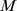 and 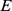 are used respectively to stand for a participant’s Wealth, Money (Cash) and Equity (No. of Stock Shares), so we can express his/her total wealth at time 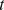 as
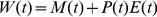
(1)where 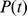 is the stock price at time 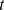. Then, we set the initial portfolio of a player 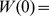 20,000 (10,000 in cash and 1,000 shares of the stock). For each round of experiments, we conduct 60 time steps, that is to say that the players could choose to trade 60 times in one round. This time scale (60 time steps) for each round of experiments would be enough. Because after 5 rounds of experiments (as we’ll show later), we have obtained enough data for our statistical analysis. And during 60 time steps, the market has well evolved to an environment where leverage effects can be captured clearly, and the participants have also shown their diversity and capability, which is important for the analysis of their wealth differences. Besides, it is also a proper time length for the real human subjects to perform one round with clear thoughts and focused attitude, since during our experiments, due to the necessary time for players’ consideration and decision, the actual time we took for one round (60 time steps) was about one hour, which is a relatively reasonable time period for participants to keep a good state. According to our experience, longer time would cause human subjects to feel tired and distracted, and even wouldn’t take the experiments seriously. This could do harm to the results from these experiments. So after trials of time control and considerations of data amount, we set it as 60.

**Figure 1:**
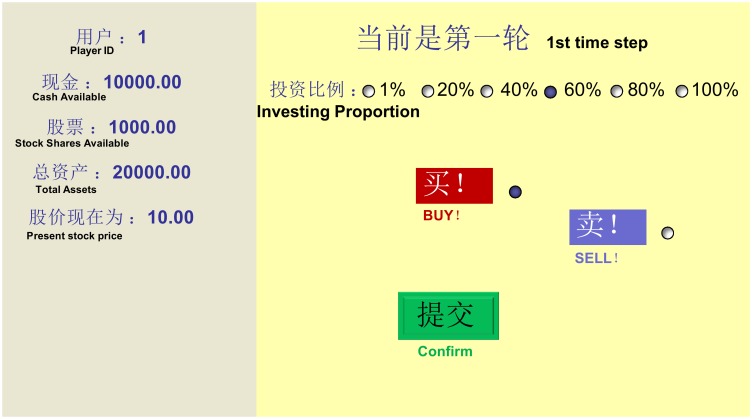
The information and operational panel of the experiments. It is in both English and Chinese. The left part is the information panel of the stock and the player, showing the financial status of both the stock and the player at each time step; and right part is the operational panel of buying and selling. With all these information that would be updated at each time step during the experiments, the players could have an overall understanding of the current situation and their historical performance, and then make their own trading decisions.

During the experiments, a panel of information is shown to each player, giving the current financial status of both the stock and the player himself/herself, as shown in Fig. 1. And when leveraged, their financial status on Fig. 1 would be updated with new information (shown in red) to show the amount of their borrowed money, and the trigger line for margin call. Besides, we give players the right to decide their order size based on their total wealth and strategies. For each time step, players decide not only their investment direction (buy or sell) but also a proportion of their total available cash or stock shares (choosing from 1%, 20%, 40%, 60%, 80%, 100%), so that the order size strategies for the players are diversified in our market, reflecting the size effect when their investments are leveraged, and the various investment strategies in the real markets.

The price changes of the stock are decided by the excess supply or demand. Here we resort to references [24], [25], the new price 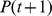 according to the supply and demand at time 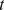 is expressed by:
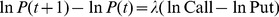
(2)


Namely,
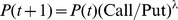
(3)


This 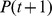 would also be the strike price for transactions. Here “Call” (or “Put”) is the total value of call/buy (or put/sell) orders, and 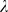 represents the market depth, which shows the sensitivity of price to supply and demand change. A lower 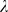 means a deeper market, since when 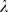 is high, the market would fluctuate heavily with large buy or sell orders, while under a lower 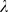 the market can be more stable when facing the same situation, indicating a deeper market. And to make the market more robust and diversified, we also introduce noise traders in the system by systematically giving one random order to buy side and another to sell side, which both consist of a small random proportion (within the range from 10% to 30%) of the value of total orders (both buy and sell) in the market at each time step.

Both of these parameter settings (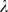 and random proportion range) are intended to offer a reasonable market behavior, especially the price movements, which would directly affect the results of wealth distribution of participants. As mentioned above, 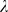 plays as the market depth to control the magnitude of price fluctuations with a certain call/put order ratio, so with a proper 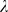, the price would fluctuate in a reasonable range. And random proportion is the size of random orders to be added in the system, since in real markets, there do exist orders that seem to be “random”. This is mostly because some investors don’t trade assets (like stocks) for profits from price movements, they just buy or sell assets for purposes like hedging or a more diversified portfolio. Besides, unpredictable investors also exist in real markets. So the introduction of random orders is necessary. However, in this work we want to mainly focus on the market participants who want to earn wealth by trading stocks so that we could reveal the leverage effect on them. That’s why we gave a dominating order value percentage of the real human players, and a random proportion range was set as 10% to 30% of the total order value. After setting this range, and according to our price determination formula above again, we could determine the value of 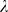.

We know that in real markets, price fluctuations could never be too outrageous. In markets like China, the price fluctuation is even strictly confined to a certain limit (10% a day, up or down). So we also want to confine our price fluctuations in a reasonable level as well. Since in our market, there could be chances that all the players choose to buy or sell, which is an extreme case but needed to be considered. And when this happens, the Call/Put ratio could be unreasonable without random orders. This is another important necessity of random orders. Let’s say all the players choose to buy (call), and the total order value is 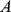. So the random call and put orders could be certain values of 10% to 30% of 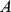. Then the extreme case for highest Call/Put ratio is that random call order is 0.3 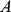 (max) while random put order is 0.1 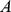 (min). Then, the maximum Call/Put ratio is 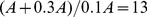. The analysis for “all sell” case is similar. So with these limits, we have tested different values of 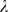 to obtain a reasonable price changing level in one time step, and set 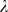 as 0.16 in our market.

### The mechanics of leverage

To make leverage available in the market, we adopt the essential ideas of leverage in the real markets: qualification demand, leverage ratio and margin calls.

- Qualification demand is the access standard for those who want to use leverage in financial markets, which usually covers a wide criteria like wealth, experience in the field, credit level, etc. The demand may vary from different countries and regions. This is to make sure that the users of leverage have the ability to take the high risk from amplified investments by leverage. In our laboratory market, we adopt a reasonable simplification by setting a demanded amount of wealth as the trigger of leverage: the initial wealth of a player 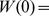
20,000, and the demanded wealth to trigger leverage 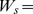
25000 (which is a 25% raise of the initial wealth). The reasonability lies in the fact that the players whose wealth exceed this demanded level are those who have better trading skills, so they could be equally treated as the qualified participants in real markets.
- Leverage ratio (denoted as LR in the context later), which is defined as the ratio of one’s total assets (borrowed assets included) to his/her own wealth, is a key element to measure the magnitude of leverage. It is also the key variable we use in this work. In real markets, this ratio is much related to another important regulative concept of leverage: margin rate, which is the least proportion of the deposit as collateral (also known as margin) a participant must have in his/her total assets account (including loans), so it controls the size of the loan, and determines the highest allowed leverage ratio. For example, if the margin rate is 50% and the margin that the borrower must offer is 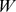, then the loan he/she can borrow at most is 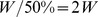. So the highest allowed LR in this case is: 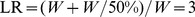. Most countries regulate the margin rate by setting its least level to control credit risk, which is various among regions. In USA, margin rate has been adjusted multiple times by the Federal Reserve, and 50% has been adopted since 1974. In China, it has also been 50% since leverage was introduced. In this work, we take the LR as the key variable in the market to study how a changing maximum leverage level affects the financial markets and its participants’ wealth distribution. And in our market, the participants will passively accept the maximum LR to invest when leverage is triggered, instead of having the right to choose a specific LR that could be lower than the maximum. This is because our main goal here is to investigate the effects of different levels of leverage ratios on wealth distribution. And by fixing this ratio as a maximum value for all the participants in each round, we were able to clearly see the differences brought by different levels of leverage regulation in the market (not by the participants’ different choices). So we decided to make this simplification from real markets. This would be a benefit from the concept “controlled experiment” as we mentioned above, because we want to separate the pure leverage effect from other factors like the different choices of human players.
- Margin call is the vital mechanics for risk control in a leveraged market. If the leveraged players fail to gain and suffer losses, how could the lending agencies ensure the safety of their loans? This is where margin call functions: under a clear threat of defaults, financial agencies will demand borrowers to add their margin and bring the LR back to a safe level to keep the borrowed funds, otherwise they’ll force borrowers to return the funds immediately by selling their stocks. This process is defined as a margin call. The lowest tolerant standard by which financial agencies will still maintain lending can be interpreted as the maintenance requirement, which is also differently ruled among the world. In our market, we take the rule in China by defining the maintenance guaranty ratio (MGR) as the standard for whether to trigger the margin call:

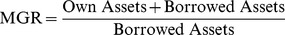
(4)We already mentioned that the least margin rate in China is 50%, so the Chinese initial MGR is 1.5. Since borrowed assets won’t be changed during the period of leveraged trading, MGR is only affected by one’s own assets. In China, the official lowest requirement of MGR is 1.3. That is to say, the Chinese financial lending agencies will allow borrowers to lose at most 40% of their own wealth (from 0.5 to 0.3 times Borrowed Assets) when they are using the highest-allowed leverage. We take this method as our margin call rule by setting a 40% loss of own assets as the trigger.
  What’s more, in order to focus on the defaults and forced returning, which happen frequently in real markets and have been blamed for creating crisis, players in our laboratory market use their whole own wealth as the initial margin to trigger leverage. So, without extra reserved margin, they will be forced to return the loans immediately when meeting the margin call. When margin calls happen, the system would help players to make sell orders automatically to return funds.

### Other details and incentive mechanics

In order to make the experimental results more general, we have done two experiments at different time and recruited different groups of participants. The first experiment was conducted on July 8, 2013, for which we recruited 22 players and studied LR = 1 and 5. Similarly, the second experiment was conducted on Sep. 27, 2013, for which we recruited 46 players and studied LR = 2, 3 and 4.

Since in our market, the stock price, which directly affects the wealth of participants, is determined by the ratio of call/put order value, not by the absolute numbers of participants or the value themselves. And as we mentioned above, the mechanics of random orders have helped us confine this ratio to a certain range of [1/13, 13]. So although the number of participants and value of call or buy orders may vary in different rounds of experiments, the price determination mechanics are always identical for the same market settings. As a result, with the comparable and consistent pricing mechanics that might affect the wealth distribution of different-size groups of participants, we can say the results are independent of absolute number of participants. This is important for the related experiments to be conducted in the future.

Next we introduce the incentive mechanism in the experiments. We all know that one important role of financial markets is to offer participants opportunities to pursue profits, so we have related the performance of players with profits to make the market closer to real ones and the experimental results more reliable. Here, “profits” could take different forms including normal money rewards. For example, in our experiments, we used two kinds of profits as rewards: cash in the 1st experiment and bonus course score in the 2nd, since a better grading in the course is also quite attractive to a student. Details are as below: In the first experiment with cash reward, we set a cash pool whose value equals the number of players multiplied by 100 Yuan, namely, 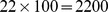 Yuan. Then we allocate this amount of money due to the weights of players’ wealth score in the whole population. This score is calculated like this.

First, we assign 70 points as a total full score for each player in the two rounds of experiments, each round corresponding to one LR, 30 points for each round and 10 points for their participating. At the end of each round, we have a wealth list of all the players, then we set the highest in the list as 30 points, all the other wealth amounts would take a proportion from the highest and then multiply 30 as its score in this round. For example, for a certain LR, if the highest wealth is 50,000 and another player gains 30,000, then his/her score for this single round is 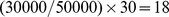. In this way, we can have the final score of each player by summing up the two performance scores and 10 points from participation. At last, we sum these scores of all the 22 players and calculate the percentage of each player’s score of the total to decide his/her final reward from the total cash pool. Say this percentage is 10%, then his/her final reward would be 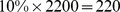 Yuan. So the outstanding players would have a good chance to achieve a reward which is much higher than the average level.

In the second experiment with course score reward, all the participants are the students from the course: Econophysics, and this experiment is part of the course in order to let the students learn how human experiments in controllable laboratory markets help in the study of econophysics. This experiment takes 15% of the full score (100) in the course. Similarly, we assign 30 points for each of the three LR round, and 10 as participation points. So the full point in this experiment is 100. But later rewarding process is a little different from Exp. 1: after ranking the total points of each player’s final score, we give the top 6 students full mark of 15, leaving 40 students to mark. Then we give them marks with four score levels: 13, 11, 9 and 7. This new system is designed from the courses grading system of Fudan University (score levels of A, B, C, D, F, etc.). Since this experiment is part of the course, this method is the most suitable way to encourage these students to perform well and improve their course knowledge and final course scores, which are just the same strong incentive as money for a student.

In the experiments, all the students have participated voluntarily, as we stated in the ethics statement, since besides the incentives of rewards like money and course scores, their active and curious attitude on scientific research is also a great motivation for them to willingly take part in the study. All the experimental data have been sent to the participants for their self study and other reference.

In this sense, we’ve built a profit-oriented environment in the laboratory market, which is equivalent to real stock markets (at least to some extent). As a result, both incentive mechanics have aroused strong motivations from participants for good performance. Meanwhile these mechanics have also effectively improved the morale and atmosphere during the experiments.

## Results and Discussion

Now we demonstrate the experimental results and the discussion on them.

### Price movements

**Figure 2:**
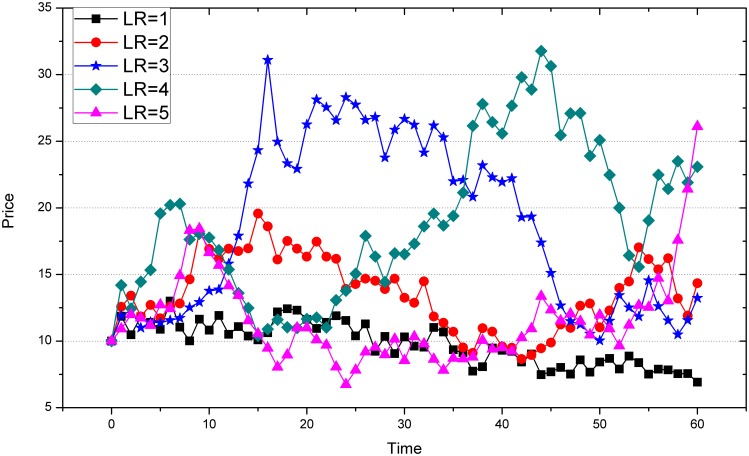
The price series of the experiments. Five rounds are represented by different colors. Leverage Ratio (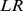) has been changed from 1 to 5.

First, we show the price series of 5 experiments in our laboratory market in Fig. 2. And see how the changing leverage ratio affects the patterns of price fluctuations, since the prices of the stock are directly linked to the wealth of players.

**Figure 3:**
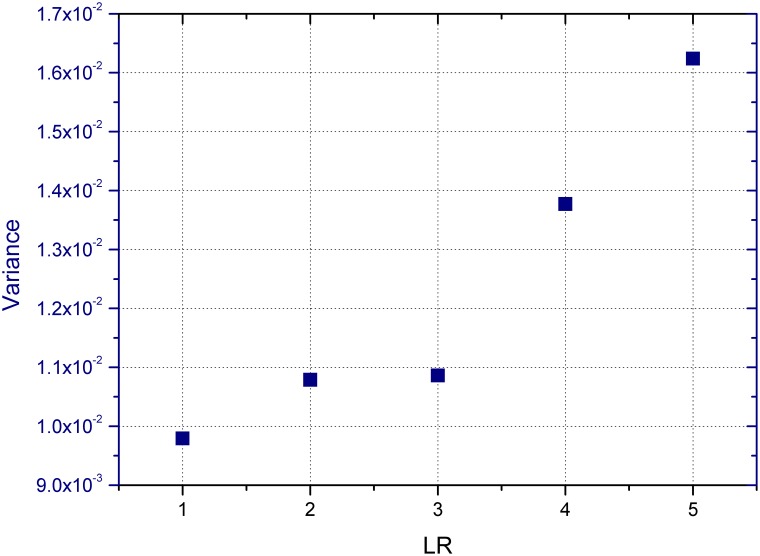
The variance trend of the price return in the experiments. It goes up with the ascending 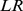, showing that a higher level of leverage brings a higher level of volatility, which is considered as the risk in the market.

Fig. 2 demonstrates a direct impression of price movements in the experiments. We can tell a clear tendency that the situation with larger LRs would have more fluctuations. More quantitatively, we can see the trend of variance of the price return, which indicates the volatility in the five experiments in Fig. 3.

As we know, the volatility of price return is the label of risk in financial markets, and here we see a clear up-trend in volatility as the LR rises, so we can tell that as the leverage becomes higher, it also brings more risk in the market, which has also been proved in other studies [5], [26], [27]. This is mainly because in the zero leverage (LR = 1) case, players could only trade with their own assets, relatively in small scales. Yet in the leveraged cases (LR = 2, 3, 4 and 5), a group of players may have the excessive amounts of money to invest when they reach the access demand, and this buying power could lead to significant upward trends in the price. And when they want to sell with large orders, or when margin calls force them to sell a lot, they would pull down the price greatly, which may cause losses to those who hold a large possession of stocks, and trigger more margin calls. The leveraging and margin call level of these experiments are shown below in Table 1.

**Table 1:** The leveraging and margin call levels of experiments.

LR	No.of Participants	LT Times	MC Times	Avg. MCs Per Capita	Avg. MCs/LTs
2	46	45	19	0.413	0.422
3	46	46	30	0.652	0.652
4	46	47	34	0.739	0.723
5	22	22	15	0.682	0.682

In this table, the LT Times (Leverage Trigger Times) means how many times leverage has been triggered in one certain round of experiment; the MC Times (Margin Call Times) means how many times margin calls have been triggered in the round, and since number of participants is different for LR  = 5, we calculated the average margin call times per person (Avg. MCs Per Capita) and average margin calls times to leverage trigger times (Avg. MCs/LTs) to make results more comparable. We can find both the margin call times and margin call possibility (MCs/LTs) are basically ascending, except for LR = 5, which is slightly lower than LR = 4. This is understandable since LR = 1 and 5 were conducted on the same day, and the participants on that day tended to be more conservative, especially when facing a much larger LR. So the average level of margin calls slightly dropped. Still, it is higher than those of LR = 2 and 3. So we can see that the average leveraging and margin call levels for different rounds have basically formed a positive relation with the leverage ratio. As we mentioned above, the rising margin calls have contributed in the rising volatility of the market, which leads to a riskier environment to participants.

Next we will find out how the changing leverage ratio and price movements are related to the pattern of wealth distribution.

### Wealth distribution

**Figure 4:**
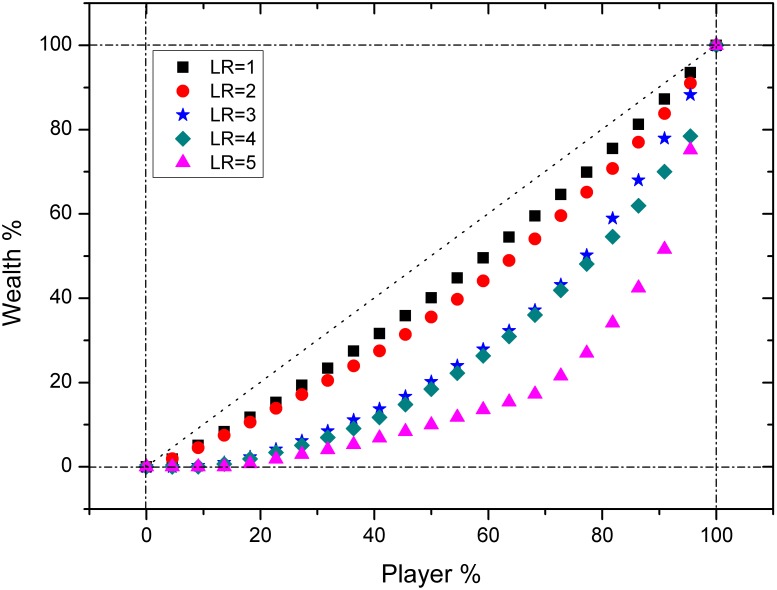
The Gini coefficient trend of the experiments with changing 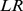. Each dot represents the percentage of total wealth (Y Axis) that a certain percentage of total players (X Axis) have. As the 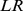 goes up, Gini coefficient (the ratio of area between the Lorenz Line and the 45 degree straight line to the whole triangle area) goes up as well, which indicates that the wealth distribution becomes more uneven.

Now let’s proceed to the core issue of this work: wealth distribution, which can be studied with Gini coefficient, as shown in Fig. 4. Gini coefficient is a measure of statistical dispersion intended to represent the wealth distribution of a population. It is usually defined mathematically based on the Lorenz curve (in colorful dots), which plots the proportion of the population’s total wealth (y axis) that is cumulatively earned by a certain proportion of the population (x axis) [28], [29]. The line at 45 degrees (in black dots) thus represents perfect equality of wealth. Then the Gini coefficient is calculated as the ratio of the area that lies between the line of equality and the Lorenz curve, over the total area under the line of equality (the right-angled triangle). So its value is confined in the range of 0 to 1. A Gini coefficient of 0 expresses perfect equality, where all members in the population have the same wealth; and a Gini coefficient of 1 expresses maximal inequality where only one person has all the wealth of the population. So generally, a higher Gini coefficient stands for a situation where the wealth is distributed more unevenly. With this key concept, we can find how the Gini coefficient changes with the varying leverage ratio in our market, as shown in Fig. 4.

From Fig. 4 we can tell that the higher LR has made the Lorenz curve more concave, which means the Gini coefficient rises with the increasing LR. This shows the wealth distribution has become more unequal. In other words, with more leverage allowed in the market, the performance of different players has become more diversified. Why does this feature happen? More volatility in the price fluctuations has raised the risk in the market, and helped in creating more and bigger gaining and losing opportunities for the players. So the players with better trading skills could use the higher leverage as a weapon for them to gain more wealth than others; while the relatively weaker performers would suffer the larger losses in more leveraged environment. As a result, the wealth distribution becomes more uneven, and the Gini coefficient rises in the meantime.

**Figure 5:**
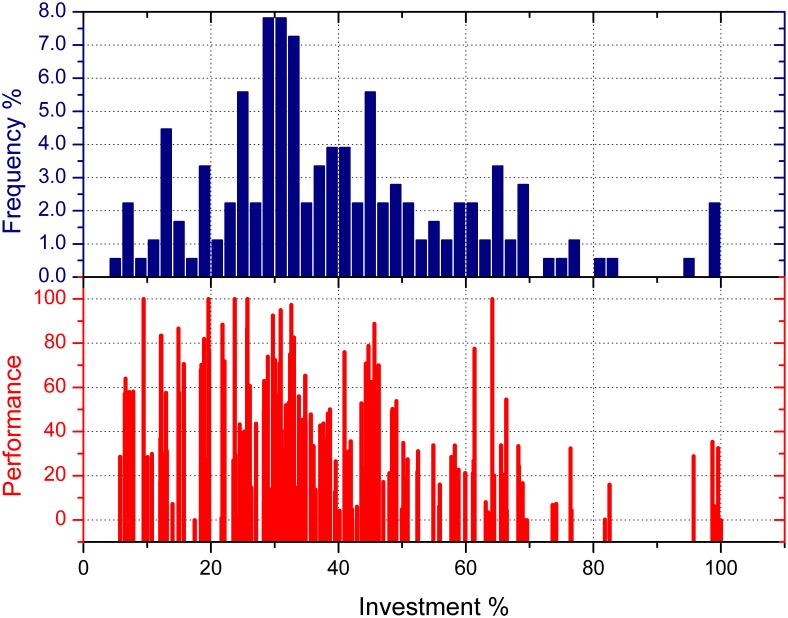
The effect of risk preference on the players’ wealth in all the experiments. The blue bar means the frequency counting number percentage of the investment proportion which lies in the certain range on the X axis; the red line means the performance score of a certain player in a certain experiment, in the scale of 0 to 100. (In order to make the results comparable, for each experiment, the highest wealth number 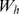 is treated as 100, and another score of a certain wealth number 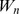 is calculated as 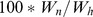).

**Figure 6:**
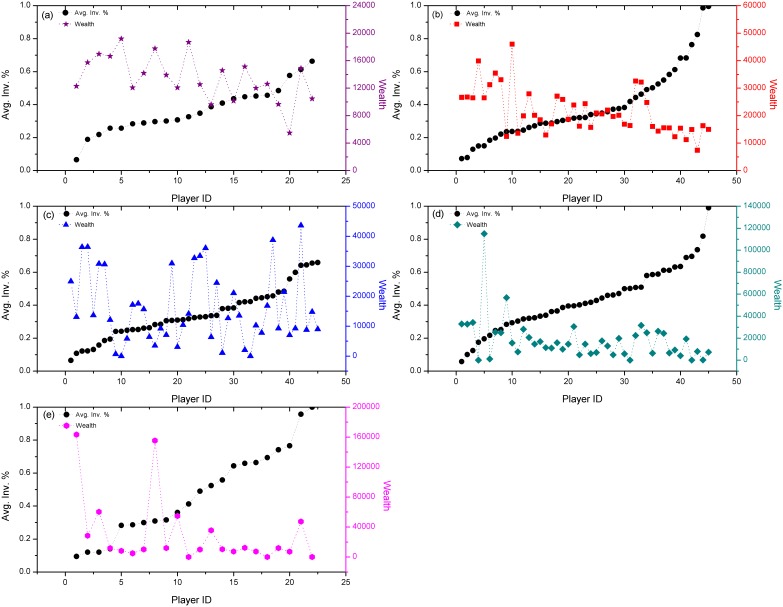
The players’ risk preference and wealth in each experiment. Black dots are the average investment proportion of each player in a certain experiment; colorful dots represent the final wealth of the player. In order to see the relation between risk preference and final wealth, Player IDs in each experiment are realigned in the order of a growing investment proportion.

Besides the reason of higher risk with leverage and different trading skills of players, the players’ preference of risk also plays a very important role in the wealth distribution mechanics. Since in our experiments, players could choose the investment proportion within 1%, 20%, 40%, 60%, 80% and 100%, and it is obvious that when choosing to invest in larger percentage, players would carry more risk. So the players who like to invest in higher percentage have higher preference for risk. Does this preference show its effect on wealth distribution? We have calculated the average investment proportion of each player in all the five experiments and try to find out the relation between their risk preference and the final wealth distribution. Results are shown in Fig. 5 and Fig. 6.

From Fig. 5, we can get an overall view of the relation between risk preference and wealth distribution in the market. Most choices of investment proportion were between the range of 20% to 50%, which means that most of the players were relatively conservative when making decisions; as a result, most of the high performance scores also fell in this range. However, the more aggressive players who chose a higher proportion mostly didn’t get a higher score. So we can tell that generally the difference of risk preference also leads to a different wealth performance, which affects Gini coefficient.

To study this phenomenon more clearly, we separately show the players’ risk preference and wealth performance in each round of experiments, as shown in Fig. 6. Black dots are the average investment proportion of each player in a certain round of experiments; colorful dots represent the final wealth of each player in the same round. We can see that basically two patterns have been formed in the five cases: for LR = 1 and 3 (Fig. 6(a, c)) the black dots lie in a relatively smaller scale (from 0 to 0.7), which means no extremely risk-chasing players occur in these two rounds of experiments. As a result, the wealth distribution has less relation with risk preference sequence, distributed more randomly; for LR = 2, 4 and 5 (Fig. 6(b, d, e)), the risk preference rates have covered the range from 0 to 1, which means that certain players have operated with high risk preference in the experiments, always in the biggest orders they can bid. Meanwhile, the wealth of players has basically declined with the growing investment proportion, leaving just very few players owning much wealth while most of others have a poor wealth performance. This is because the existence of extremely aggressive players has helped in increasing the volatility and unpredictability of the market. So besides the different trading skills mentioned above, the diversified risk preference of players has also contributed to the risk in the market. Especially in the environment of leverage where the population is exposed to higher risk, this effect of diversity has been amplified, including both the different skills and risk preference of individuals, and then it eventually shows in the form of a more diversified wealth distribution. So to sum up, the combined force of leverage mechanics and diversity of the population leads to a higher Gini coefficient, a.k.a. a more unequal wealth distribution.

## Conclusions

In this paper we have built a controllable laboratory market where participants can trade stocks, and we have studied the effects of leverage in the market, especially on the wealth distribution of participants. By conducting human experiments we have drawn the conclusion that more leverage leads to a higher Gini coefficient in the system, which means the wealth distribution becomes more unequal. This is because of the ascending risk brought by the leverage mechanics, and the different trading skills and risk preference of the population. The work is insightful for the effects of leverage and its related regulations, and also shows the reasonability and capability of the method of controllable laboratory markets, which can be further utilized in multiple fields in both econophysics and economics.
